# Supplementary material for: MP Allosterically Activates AMPK to Enhance ABCA1 Stability by Retarding the Calpain-Mediated Degradation Pathway
Source: Int J Mol Sci. 2023 Dec 8;24(24):17280. doi: 10.3390/ijms242417280 (PMC10743971; doi:10.3390/ijms242417280)
Supplement: Supplementary file 1 [file ijms-24-17280-s001.zip › Supplementary materials.pdf]

Supplementary Methods and Data for

MP allosterically activates AMPK to enhance ABCA1 stability by retarding the calpain-mediated degradation pathway

Hui Li [a], Mingchao Wang [a], Kai Qu [a], Ruiming Xu [a], Haibo Zhu [a, *]

[a] State Key Laboratory for Bioactive Substances and Functions of Natural Medicines, Beijing Key Laboratory of New Drug Mechanisms and Pharmacological Evaluation Study, Institute of Materia Medica, Chinese Academy of Medical Sciences and Peking Union Medical College, Xian Nong Tan Street 1, Xicheng District, Beijing 100050, China.

*Corresponding author.

E-mail addresses: zhuhaibo@imm.ac.cn (Haibo Zhu)

Supplementary Methods

1.1 Chemicals and Instrumentation

DL-dithiothreitol (DTT), iodoacetamide (IAM), formic acid (FA), acetonitrile (ACN), methanol, were purchased from Sigma (St. Louis, MO, USA), Protease was purchased from Promega (Madison, WI, USA). Ultrapure water was prepared from a Millipore purification system (Billerica, MA, USA). An Easy-nLC 1000 system coupled with a Orbitrap Exploris 240 Mass Spectrometer (Thermo Fisher Scientific, USA) with an ESI nanospray source.

1.2 Sample Preparation

1.2.1 Digestion

a) Cut three excised gel slices from each gel into 1 mm^3 cubes and transfer the gel cubes to a 1.5-mL microcentrifuge tube. Centrifuge the tube for 10-30 min to spin the gel slices to the bottom of the tube. Add 500 μL of 50 mM ammonium bicarbonate/acetonitrile (1:1, v/v) solution and wash until Coomassie blue disappear. Remove the supernatant.

b) Add 100 μL of acetonitrile and incubate for 30 min. The gel pieces should become opaque and stick together.

c) Remove the acetonitrile using a pipettor with a clean pipette tip. Rehydrate the gel slices in 10 mM DTT/50 mM ammonium bicarbonate. Add enough solution to completely cover the gel slices. Incubate at 56°C for 1 hour.

d) Remove the acetonitrile using a pipettor with a clean pipette tip. Add the 55 mM IAM/50 mM ammonium bicarbonate to completely cover the gel slices. Incubate for 1 hour at room temperature in the dark. Remove the supernatant.

e) Add 100 μL of 50 mM ammonium bicarbonate/acetonitrile (1:1, v/v) solution, and remove the supernatant. Add 100 μL of acetonitrile and incubate for 30 min. The gel pieces should become opaque and stick together. Remove the acetonitrile solution.

f) Add just enough enzyme digestion solution to cover the gel slices. Incubate the gel pieces on ice for 40 min. Add more digestion solution if all the initial solution is absorbed by the gel pieces.

g) Add 5-10 μL of 50 mM ammonium bicarbonate to keep the gel pieces wet during enzymatic digestion. Incubate overnight at 37°C.

h) Add extraction solution (5% TFA-50% ACN-45% ddH2O) 100 μL / tube, 1 hour at 37 °C water bath, sonicate for 5 min, centrifuge for 5 min, transfer the extract to a fresh 1.5 mL microcentrifuge tube, the extraction was repeated once.

i) Lyophilize the extracted peptides to near dryness.

1.3 Nano LC-MS/MS Analysis

1.3.1 nanoLC

Nanoflow UPLC: Easy-nLC 1000 system (ThermoFisher Scientific, USA).

Nanocolumn: 150 μm×15 cm in-house made column packed with Acclaim PepMap

RPLC C18 (1.9 μm, 100 Å, Dr. Maisch GmbH, Germany).

Loaded sample volume: 5 μL.

Mobile phase, A: 0.1% formic acid in water; B: 0.1% formic acid in water with 80% acetonitrile.

Total flow rate: 600 nL/min.

LC linear gradient: from 4% to 8% B for 2 min, from 8% to 28% B for 33 min, from 28% to 40% B for 20 min and from 40% to 95% B for 10 min.

1.3.2 Mass spectrometry

Orbitrap Exploris 240 Mass Spectrometer (Thermo Fisher Scientific, USA)

Spray voltage: 2.2 kV

Capillary temperature: 270°C

MS parameters:

MS resolution: 70000 at 400 m/z

MS precursor m/z range: 300.0-1800.0

MS/MS parameters:

Activation Type: HCD

Normalized Coll. Energy: 30.0

Activation Time: 66.000

Data dependent MS/MS: up to top 20 most intense peptide ions from the preview scan in the Orbitrap.

1.4 Data analysis

The raw MS files were analyzed and searched against target protein database based on the species of the samples using Byonic. The parameters were set as follows: the protein modifications were carbamidomethylation (C) (fixed), oxidation (M) (variable), Phospho (S,T,Y) (variable), the enzyme specificity was set to trypsin or chymotrypsin; the maximum missed cleavages were set to 3; the precursor ion mass tolerance was set to 20 ppm, and MS/MS tolerance was 0.02 Da. Only high confident identified peptides were chosen for downstream protein identification analysis.

Supplementary data

Total Ions Chromatogram

BTP20230705-Control-C:

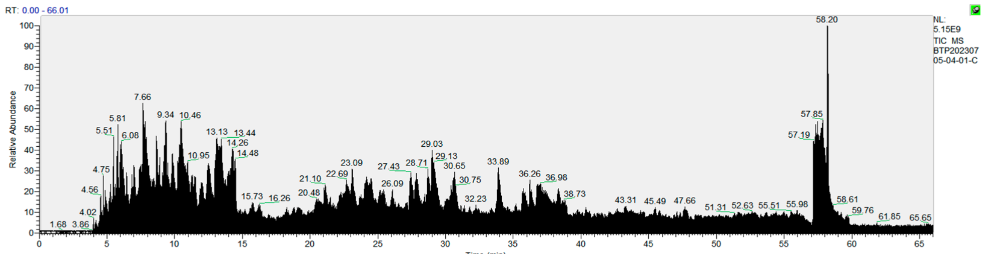

BTP20230705- Control-T:

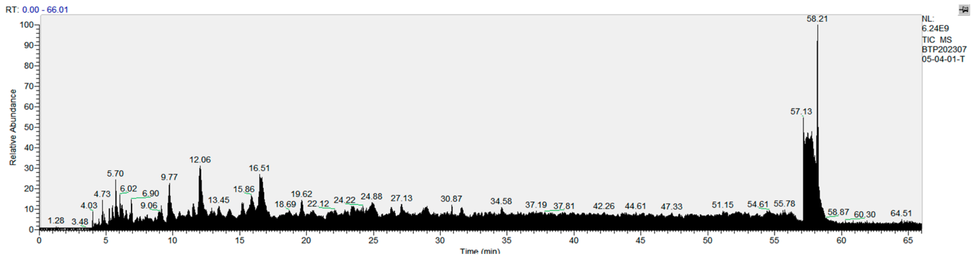

BTP20230705-MP-C:

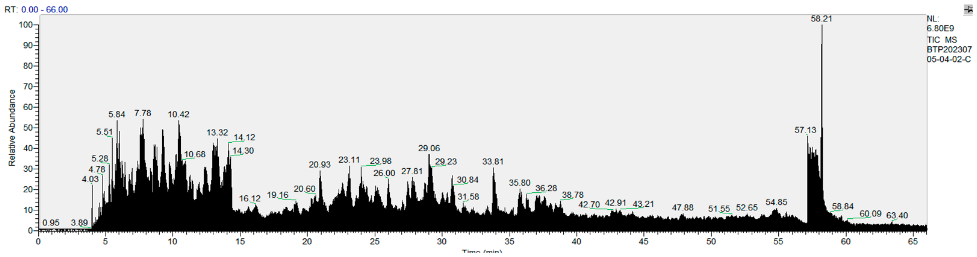

BTP20230705-MP-T:

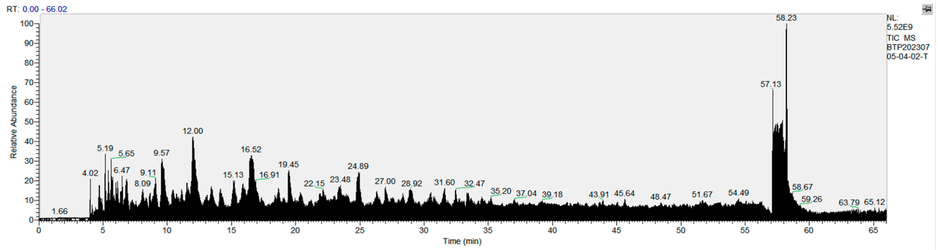

BTP20230705-Ionomycin-C:

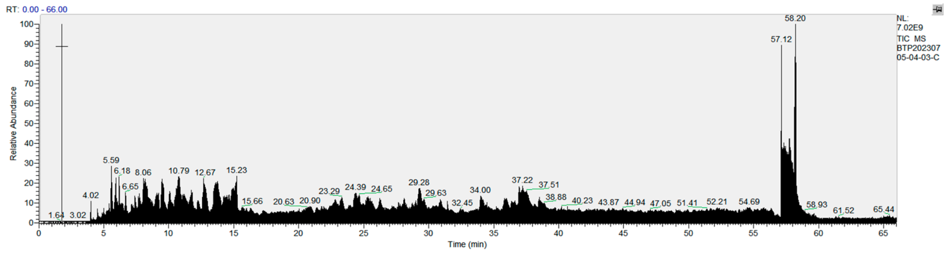

BTP20230705-Ionomycin-T:

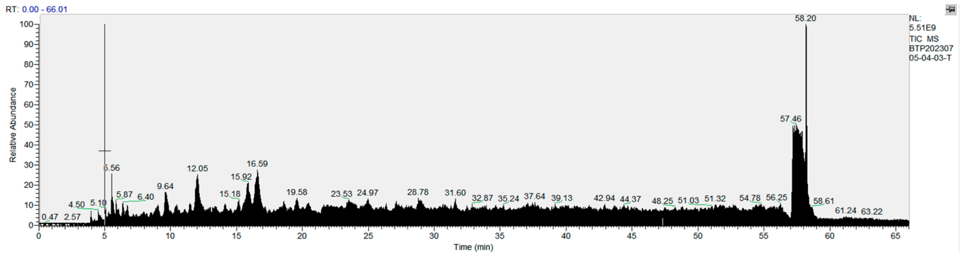

BTP20230705-MP+Ionomycin-C:

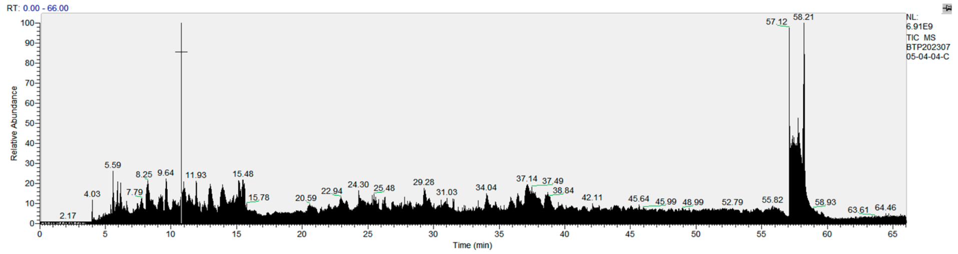

BTP20230705-MP+Ionomycin-T:

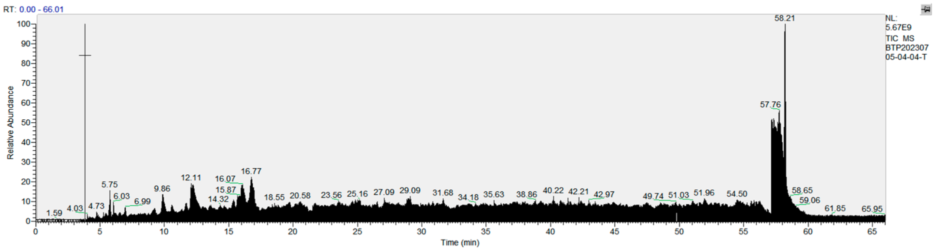

RT: 0.00 - 66.01
NL: 5.67E9
TIC MS
BTP202307
05-04-04-T
Relative Abundance
Time (min)
58.21
57.76
16.77
12.11
16.07
15.87
14.32
9.86
5.75
6.03
6.99
4.73
4.03
1.59
18.55
20.58
23.56
25.16
27.09
29.09
31.68
34.18
35.63
38.86
40.22
42.21
42.97
49.74
51.03
51.96
54.50
58.65
59.06
61.85
65.95
